# Recognition of *Bungarus multicinctus* Venom by a DNA Aptamer against β-Bungarotoxin

**DOI:** 10.1371/journal.pone.0105404

**Published:** 2014-08-21

**Authors:** Fengping Ye, Ying Zheng, Xi Wang, Xiaolong Tan, Tao Zhang, Wenwen Xin, Jie Wang, Yong Huang, Quanshui Fan, Jinglin Wang

**Affiliations:** 1 State Key Laboratory of Pathogen and Biosecurity, Institute of Microbiology and Epidemiology, Beijing, China; 2 Institute of Military Medicine, Chengdu Military Region's Center for Disease Control & Prevention, Kunming, China; Imperial College London, United Kingdom

## Abstract

Antibody-based technology is the main method for diagnosis and treatment of snake bite envenoming currently. However, the development of an antibody, polyclonal or monoclonal, is a complicated and costly procedure. Aptamers are single stranded oligonucleotides that recognize specific targets such as proteins and have shown great potential over the years as diagnostic and therapeutic agents. In contrast to antibodies, aptamers can be selected in vitro without immunization of animals, and synthesized chemically with extreme accuracy, low cost and high degree of purity. In this study we firstly report on the identification of DNA aptamers that bind to β-bungarotoxin (β-BuTx), a neurotoxin from the venom of *Bungarus multicinctus*. A plate-SELEX method was used for the selection of β-BuTx specific aptamers. After 10 rounds of selection, four aptamer candidates were obtained, with the dissociation constant ranged from 65.9 nM to 995 nM measured by fluorescence spectroscopy. Competitive binding assays using both the fluorescently labeled and unlabeled aptamers revealed that the four aptamers bound to the same binding site of β-BuTx. The best binder, βB-1, bound specifically to β-BuTx, but not to BSA, casein or α-Bungarotoxin. Moreover, electrophoretic mobility shift assay and enzyme-linked aptamer assay demonstrated that βB-1 could discriminate *B. multicinctus* venom from other snake venoms tested. The results suggest that aptamer βB-1 can serve as a useful tool for the design and development of drugs and diagnostic tests for β-BuTx poisoning and *B. multicinctus* bites.

## Introduction

Snakebite is a major public health issue that affects residents of rural communities living in tropical and subtropical countries [1]. It was estimated that, globally, between 1.2 million and 5.5 million people are bitten by snakes annually, resulting in about 20,000–125,000 deaths, and 400,000 permanent disabilities [2]–[4]. The administration of animal-derived antivenoms is the mainstay treatment of snakebite envenoming. Conventional antivenoms are prepared from sera of large animals, usually horses or sheep, hyperimmunized with relevant snake venoms. After collection of blood or plasma, the plasma is fractionated to extract and purify the active immunoglobulin or immunoglobulin fragments (F(ab')_2_), and then the antivenom is freeze-dried for conservation and transportation. Due to its complex and time consuming process of production, antivenom is so expensive that many people actually cannot afford the high costs. Moreover, antivenom therapy of snakebite has long been associated with a high incidence of adverse reactions such as pruritus, urticaria and potentially fatal anaphylaxis [5], [6]. The complexity of the production of antivenoms, the decreasing number of producers and the fragility of the production systems in developing countries jeopardize the availability of effective antivenoms in Africa, Asia, the Middle East and South America [7].

Identification of snakebite is also difficult as lack of a rapid and reliable diagnostic test. Polyvalent antivenom is often used when the snakebites can not be identified, theoretically increasing the risk of late antivenom reactions because a higher dose of foreign protein is administered [5]. Current efforts in the diagnosis of snake envenomation have also been mainly focused on the development of antibody-based immunoassays. Snake venoms are highly complex mixtures that tend to have many common antigens among snakes from the same or even different genera [8], [9]. Direct use of crude antibody in immunoassays may have low specificity in species detection of snakebites. Monoclonal antibodies against a single venom component or species-specific polyclonal antibodies produced by passing the crude antisera through the medium coupled with heterologous venoms were used to reduce cross reactivities [8], [10], [11].

A new class of short single-stranded oligonucleotides (RNA or DNA) termed aptamers has quickly emerged as a novel and powerful class of ligands with excellent potential for diagnostic and therapeutic applications [12], [13]. Aptamers are selected *in vitro* through the systematic evolution of ligands by exponential enrichment (SELEX) process [14], [15], that have high affinity and specificity to a wide range of targets, such as small molecules, proteins, or whole cells [16]–[18]. As termed “chemical antibodies”, aptamers have several advantages over their counterparts. They can be selected in vitro from random DNA or RNA libraries without immunization of animals, and synthesized chemically in a readily scalable process with extreme accuracy, low cost and high degree of purity. They display low to no immunogenicity even administered in high doses. Aptamers can also be easily modified by various dyes or functional groups during chemical synthesis and immobilized on transducing device widely used in biosensors.

The many-branded krait *Bungarus multicinctus* is widely distributed throughout Southeast Asia. The snake possesses extremely toxic venom, one of the most potent of any land snake. Bitten by *B. multicinctus* does not give rise to swelling or necrosis at the site of the bite, but cause severe neuromuscular blockade, resulting in respiratory failure and fatality [19]–[21]. *B. multicinctus* was responsible for 8.12% of snakebites and the highest mortality in China [22]. The venom of *B. multicinctus* consists of both pre- and postsynaptic neurotoxins, including α-, β-, γ- and κ-bungarotoxin. Although α-bungarotoxin (α-BuTx) is the major component (61%) of the *B. multicinctus* venom [23], β-bungarotoxin (β-BuTx) is more important and lethal than α-BuTx from a toxicological or clinical perspective [24]. β-BuTx constitutes >20% of the protein content of the crude venom [25]. The minimum lethal dose of β-BuTx by intraperitoneal injection of mice is 0.01 µg/g [26], while that of α-BuTx is 0.139 µg/g [27]. β-BuTx is a presynaptic neurotoxin with a molecular weight of 21.8 kDa, consisting of two dissimilar polypeptide chains, the phospholipase A2 subunit A chain (∼14 kDa) and the non-phospholipase A2 subunit B chain (∼7 kDa), cross-linked by an interchain disulfide bond [26], [28], [29]. β-BuTx presynaptically inhibits acetylcholine release in both the peripheral and central nervous systems [30].

Until now, several aptamers targeting biotoxins have been selected [31], including bacterial toxins [32], mycotoxins [33] and plant toxins [34]. However, few aptamers are developed against snake venom components. The only aptamer against snake venom component developed to the present is a DNA aptamer against α-BuTx [35]. But the binding affinity of this aptamer is relatively low, with a dissociation constant of 7.58 µM, thus may limit its use in biological applications. Here in the present study we aim at generating aptamers with high affinity and specificity against β-BuTx that can be applied to the diagnosis and/or therapy of snakebites. By using a plate-SELEX process, aptamers were selected from a random ssDNA library. The dissociation constant values were determined by fluorescence anisotropy measurements and found to be in the range of 10^−8^ M. The aptamers bind with high affinity only to β-BuTx and β-BuTx containing *B. multicinctus* venom, but not to α-BuTx or other snake venoms tested. The ability of the aptamers to specifically recognize *B. multicinctus* venom offering the potential for application to the diagnosis of *B. multicinctus* bites.

## Materials and Methods

### Reagengts

The 80-nt single-stranded DNA (ssDNA) library contained a randomized region of 40-nt central region flanked by two conserved 20-nt primer hybridization regions in each end (5′-AGCAGCACAGAGGTCAGATG-N40-CCTATGCGTGCTACCGTGAA-3′). The fluorescein labeled forward primer (F-fam: 5′-FAM-AGCAGCACAGAGGTCAGATG-3′) and the phosphate labeled reverse primer (R-pho: 5′-phosphate-TTCACGGTAGCACGCATAGG-3′) were used in PCR to obtain double-stranded DNA (dsDNA). The library and all primers were synthesized by Sangon Biotech (Shanghai, China). Lyophilized crude venoms were obtained from a serpentarium from Jiangxi Province, China. BSA and casein were purchased from the Sigma–Aldrich Co. (USA). β-BuTx was from Santa Cruz (USA), and α-BuTx was from Abcam (UK). Premix Taq polymerase, pMD19-T vector, and DH5α competent cells were purchased from TaKaRa Biotechnology Co. Ltd. (Dalian, China). All the other reagents used for chemical and biological characterization were of analytical grade.

### SELEX Procedure

To select aptamers directed to β-BuTx, 10 rounds of plate SELEX were carried out. A 96-well ELISA polystyrene plate (Corning, USA) was coated at 4°C overnight with 100 µL of 10 µg/mL β-BuTx in 0.05 M carbonate-bicarbonate buffer (1.59 g/L Na_2_CO_3_ and 2.93 g/L NaHCO_3_, pH 9.6). After coated, the wells were blocked at room temperature (RT) for 2 h using binding buffer (20 mM HEPES, pH 7.4, 150 mM NaCl, 5 mM KCl, 2 mM MgCl_2_ and 2 mM CaCl_2_) containing 1 mg/mL BSA for rounds 1–5. For rounds 6–10, wells were blocked with binding buffer containing 1 mg/mL casein. This switch of blocking agent was intended to decrease background binding of aptamer pools to the plate surface [36]. The ssDNA library or aptamer pool in binding buffer was heated at 90°C for 10 min in a thermal cycler, immediately left to cool on ice for another 10 min, and then kept at RT for 10 min. For the first round of selection, 1 nmol of ssDNA library (∼10^14^ different sequences) was incubated with β-BuTx coated wells at RT for 1 h. For the next 9 rounds of selection, ssDNA pool used for incubation was gradually decreased to 50 pmol. After incubation, the supernatant was discarded and the wells were washed 6 times with 200 µL of washing buffer (binding buffer containing 0.05% Tween 20), and further washed 3 times with 200 µL of ddH_2_O. To elute the bound ssDNA, 100 µL of ddH_2_O was added to the wells (each well was separated from the strip and sealed with silver paper) and placed in a Thermomixer Compact (Eppendorf, Germany) at temperature of 95°C for 10 min. The supernatant was collected and served as the template for the amplification of β-BuTx-bound ssDNA.

During every round of selection, a pilot PCR was first performed to determine the optimal cycle number of amplification as described by Lou et al. [37]. Then dsDNA was amplified under the optimal cycles. One hundred microliters of the PCR mixture contained 50 µL Premix Taq solution, 4 µL template, 1 µL of 10 µM fluorescein labeled forward primer F-fam, 1 µL of 10 µM phosphate labeled reverse primer R-pho, and 44 µL of ddH_2_O. PCRs conditions were as follows: preincubation at 94°C for 5 min; optical number of cycles consisting of denaturation at 94°C for 0.5 min, annealing at 56°C for 0.5 min and extension at 72°C for 0.5 min; and final extension at 72°C for 10 min. The dsDNA PCR product was digested by λ exonuclease (NEB, UK) for 2 h at 37°C to generate ssDNA, according to the manufacturer's protocol. Reaction was stopped by 10 min incubation at 75°C. ssDNA was precipitated with dehydrated alcohol and washed with 70% alcohol. After centrifugation, the pellet was dissolved in binding buffer and used as the enriched library for the next selection round. The concentration of ssDNA was evaluated by electrophoretic analysis [38]. Each sample (10 µL) was compared with 10 µl of ssDNA library of known concentration (20, 40, 60, 80, 100 nM) using agarose gel electrophoresis. Electrophoretic analysis of the band intensities was performed using Image J program.

After 10 rounds of selection, the collected aptamer pool was PCR amplified with unlabeled forward and reverse primers. The dsDNA product was subjected to 3% agarose gel and the corresponding strand was excised and purified using the QIAEX II Gel Extraction kit (Qiagen, Germany) according to the manufacturer's instructions. Purified PCR product was ligated into the pMD19-T vector and transformed to DH5α competent cells by heat shock. Forty-five colonies were randomly picked and sequenced by Sangon Biotech (Shanghai, China). Secondary structure analysis was performed with Mfold software (http://mfold.rit.albany.edu/?q=mfold/DNA-Folding-Form).

### Nitrocellulose filter binding assay

The 0.22-µm HAWP nitrocellulose filters (Millipore) were pre–treated with alkali to reduce the non–specific adsorption of nucleic acids as described [39]. The filters were soaked in 0.5 M KOH at RT for 20 min, then washed extensively with ddH_2_O. After washing for 45 min in binding buffer by shaking, the filters were stored in new buffer at 4°C.

For measuring the aptamer pool binding, 1 mL of 10 nM fluorescein-labelled aptamer pool in binding buffer was heated to 90°C for 10 min and rapidly put on ice for 10 min, then incubated 10 min at RT. β-BuTx was then added to a final concentration of 100 nM and incubated at RT for 40 min. Controls did not contain β-BuTx. Afterwards, the mixture was vacuum filtered over a pretreated nitrocellulose, and washed with 1 mL of binding buffer. Total volume of 2 mL of aptamer DNA eluted from the filter was determined by fluorescence measurement using a Perkin-Elmer LS55 Fluorescence Spectrophotometer. The excitation wavelength was set at 492 nm and the emission wavelength at 518 nm. Slits for both the excitation and the emission were set at 10 nm, and the integration time was set at 10 sec. The fluorescence of sample elution subtracted from control elution gave the fluorescence of β-BuTx-bound ssDNA that maintained on the filter.

To measure the aptamer binding to other relative proteins, fluorescein labeled aptamer was chemically synthesized after sequencing. Ten nM of fluorescein labeled aptamer was mixed with 100 nM of β-BuTx, BSA, α-BuTx and 2.1 µg/mL of casein in binding buffer, respectively, and then subjected to nitrocellulose filter binding assay using the same above procedure.

### Fluorescence anisotropy measurements

Fluorescence anisotropy measurements were performed on Perkin-Elmer LS55 Fluorescence Spectrophotometer with excitation and emission polarizers in L-format, and the settings were the same as above. The G factor was automatically calculated by the instrument. All experiments were carried out at RT. The concentration of fluorescein labeled aptamers was 2 nM (in binding buffer) and the total volume was 2 mL. Different concentrations of β-BuTx were titrated into the aptamer solution and incubated for 2 min, and then the fluorescence anisotropy values were measured. Four anisotropy measurements were taken each time, and the experiments were repeated three times. The relative standard deviation was <3% for all the measurements. The change in anisotropy was the average anisotropy of the initial fluorescein labeled aptamer subtracted from the average anisotropy value at each β-BuTx concentration. The dissociation constant (K_d_) was determined by non-linear regression analysis with software SigmaPlot 10.0 by using a single-site ligand binding model.

For competitive displacement experiments, 2 nM of fluorescein labeled aptamer (βB-1, βB-20, βB-19 or βB-32) and 288 nM of β-BuTx were premixed in 2 mL of binding buffer and following that, titration experiments were conducted with increasing amount of unlabeled βB-1 respectively. Fluorescence anisotropy values were measured as mentioned above. As control, the curve generated by fluorescein-labeled βB-1 against unlabeled starting ssDNA library was also measured.

For specificity assays, the anisotropy of 2 nM of fluorescein labeled aptamer βB-1 in 2 mL of binding buffer was measured. And then, 50 nM of β-BuTx or 500 nM of BSA and α-BuTx, or 10 µg/mL of casein was added, respectively. The corresponding changes of anisotropy were recorded.

### Electrophoretic Mobility Shift Assay (EMSA)

Crude venoms of *B. multicinctus*, *B. fasciatus*, *Deinagkistrodon acutus*, *Gloydius brevicaudus* and *Naja Naja Atra* were resolved in binding buffer, and centrifuged at 12 000 g for 10 min, and the supernatant was collected and stored at −20°C. Fluorescein labeled aptamer βB-1 (100 nM) was mixed with β-BuTx (240, 480 or 960 nM) or *B. multicinctus* venom (15, 30 or 50 µg/mL) in binding buffer of 20 µL total volume, respectively. The binding reaction was carried out at RT for 40 min and then analyzed by 1% agarose gel containing 0.5 mg/ml of ethidium bromide. Binding reaction was also performed by using 100 nM of βB-1 and 50 µg/mL of the five snake venoms mentioned above.

### Enzyme-linked aptamer assay (ELAA)

Aptamer DNA was labeled with biotin during chemical synthesis. BSA, casein, α-BuTx and different crude snake venoms were diluted to 10 µg/mL in 0.05 M carbonate-bicarbonate buffer, pH 9.6, and incubated in an ELISA polystyrene plate strip (Corning, USA) overnight at 4°C to allow plate binding. Plate wells were washed three times with 200 µL of washing buffer, and then blocked at RT for 2 h with 100 µL of 1 mg/mL BSA in binding buffer. After washing three times in washing buffer, 100 µL of 10 pM biotin-labeled aptamer was added to individual wells and incubated at RT for 40 min. After the above procedures, individual wells were washed three times with washing buffer to remove unbound ssDNA. Then 100 µL of a 1∶1000 dilution of horseradish peroxidase (HRP)-labeled Streptavidin (Solarbio, China) in binding buffer was added to the wells. Following a 40-min incubation at RT, the wells were washed three times and 100 µL of freshly prepared substrate OPD solution (Sigma, USA) was added according to the manufacturer's instruction. The wells were kept in dark at RT for 15 min and 50 µL of 2 M H_2_SO_4_ was added to each well to stop the reaction.

## Results and Discussion

### SELEX Selection

Starting from a library of ∼10^14^ different sequences of oligodeoxyribonucleotides, we selected aptamers binding to β-BuTx, using plate SELEX technique. The critical step in SELEX is the partitioning of the target-bound oligonucleotides from the unbound ones. There are several ways to carry out the separation process, such as using of nitrocellulose membrane [40], affinity column [41], magnetic beads [42] or capillary electrophoresis [43]. Immobilization of target protein on a 96-well microtiter plate provides a simple and rapid way to separate aptamer-target complex from free DNAs. We first adsorbed β-BuTx to polystyrene 96-well plates, and then incubated with the ssDNA library pool containing a 40-nucleotide random sequence region. Following washes by washing buffer and ddH_2_O to remove unbound ssDNA, the β-BuTx-bound aptamers were eluted by heat shock at 95°C with ddH_2_O. High concentration of guanidinium isothiocyanate buffer was often used in elution of aptamers from the plate, and a phenol-chloroform extraction and an alcohol precipitation procedure was performed to purify the ssDNA [44], [45]. We found that a heat shock with ddH_2_O was sufficient to elute bound ssDNA from the plate, and it could be directly served as the template in the following PCR reactions without any purification steps. There was probably some residual β-BuTx in the elution solution, but the β-BuTx was denatured, so we thought it would have no significant interference in the PCR amplifications.

**Figure 1:**
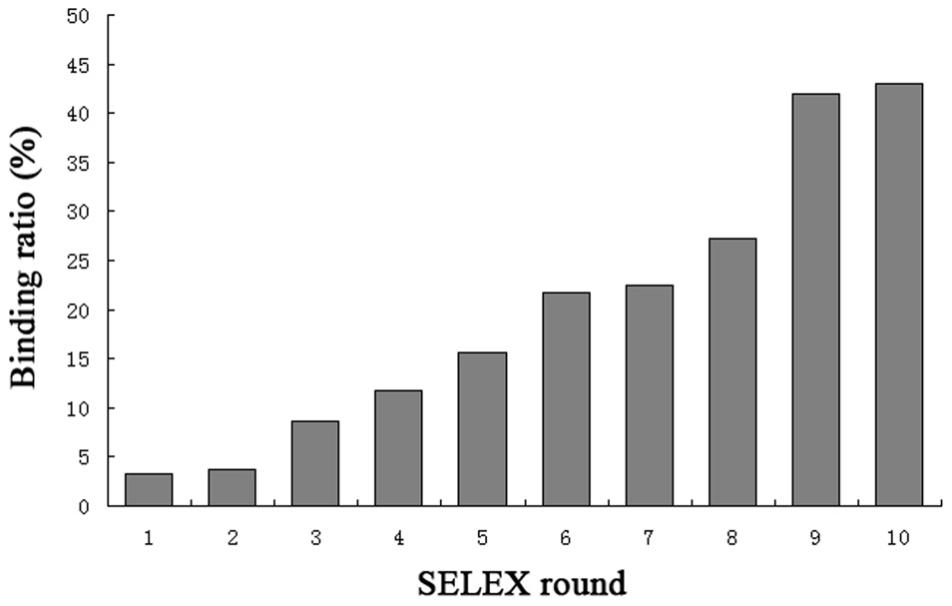
Binding affinity of ssDNA pools of each round of selection. Ten nM of ssDNA pool was incubated with 100 nM of β-BuTx and the ratio of ssDNA bound to β-BuTx was determined by nitrocellulose filter binding assay.

The selected ssDNA pool was amplified using pilot PCR in ranges of 14 to 26 PCR cycles in order to optimize the amplification cycle number under which neither shorter nor longer PCR byproducts appeared (Figure S1). The forward and reverse primers were fluorescein and phosphate labeled at 5′ end, respectively. Then the dsDNA PCR product was amplified under the optimal cycles and subjected to λ exonuclease digestion to produce fluorescein labeled ssDNA. Phosphorylated strands of DNA are selectively digested by the enzyme, which has greatly reduced activity on ssDNA and non-phosphorylated DNA. The generated ssDNA was used for the next cycle of SELEX. The amount of β-BuTx added to coat the plate wells in each round was fixed to 1 µg, and the amount of input ssDNA was gradually decreased (Table S1). BSA was used to block the wells in rounds 1-5 and then casein replaced BSA in rounds 6–10. The switch of blocking agent could dramatically decrease aptamer pool binding to the plate surface [36], so negative selections using uncoated wells were not included in our selection process. During the selection, the selection progress was monitored by determining the binding capacity of the ssDNA pool to β-BuTx via nitrocellulose filter binding assay. Nitrocellulose filter membrane possesses long pores that are nucleic acid permeable but retain proteins as small as 2 kDa by hydrophobic adsorption [46]. The ssDNA-protein complex will retain on the filter when passing through the membrane. As shown in Figure 1, with increasing numbers of selection rounds, the affinity of the DNA for the target increased accordingly, and totally ten rounds of selection were performed.

### Sequencing and structural analysis

The elution product of the 10^th^ round selection was amplified with unlabeled forward and reverse primers. After purification, the dsDNA was ligated into pMD19-T clone vector and transformed to *E. coli* cells. Forty-five positive colonies were randomly selected for sequencing, and 37 sequences were returned. Sequence comparisons revealed that only four individual binding species remained in the population, and the sequences were designated as βB-1, βB-19, βB-20 and βB-32, respectively (Table 1). One of these individuals, βB-1, dominated the selection and constituted more than 70% of the clones, while three other sequences together represented 27% of the clones. The length of variable region of aptamer βB-20 (51 nt) was significantly longer than the 40 nt randomized region of ssDNA library. Since longer or shorter byproducts usually appeared during the PCR reactions, maybe some longer products, but not long enough to be eliminated by gel purification, existed in the final aptamer pool.

**Table 1:** Sequences of selected aptamers and their dissociation constants (K_d_).

Name	Sequence of variable region	Percentage of total sequences(%)	K_d_ (nM)
βB-1	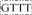 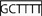 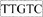 GCTTTT 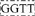 CGTTCTGCCTCTATCT 40 nt	73.0	65.9
βB-20	ATTAGTCAT 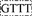 GT 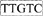 TG 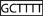 TG 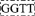 TGTGCAGTATTATGAAC 51 nt	8.1	83.8
βB-19	TTTGGTGTGGATCCTGAACATTTATATTCTTTCGTTTTTT 40 nt	16.2	530
βB-32	GCAATGCACCTTTGTCTCTTATAGTTTATT TTTTGCCTT 39 nt	2.7	995

The boxed sequences were constant regions between aptamers βB-1 and βB-20.

A further analysis with these sequences revealed a few conserved short sequences between βB-1 and βB-20 (Table 1). The secondary structures of the four sequences were evaluated at 25°C, 150 mM NaCl, and 2 mM MgCl_2_ using the Mfold software (Figure S2). All predicted secondary structures have stem-loop architectures. More notably, aptamers βB-1 and βB-20 share similar types of stems and hairpin loops, and the conserved sequences are involved in the formation of stems and loops (Figure S2).

### Binding characterization of selected aptamers

**Figure 2:**
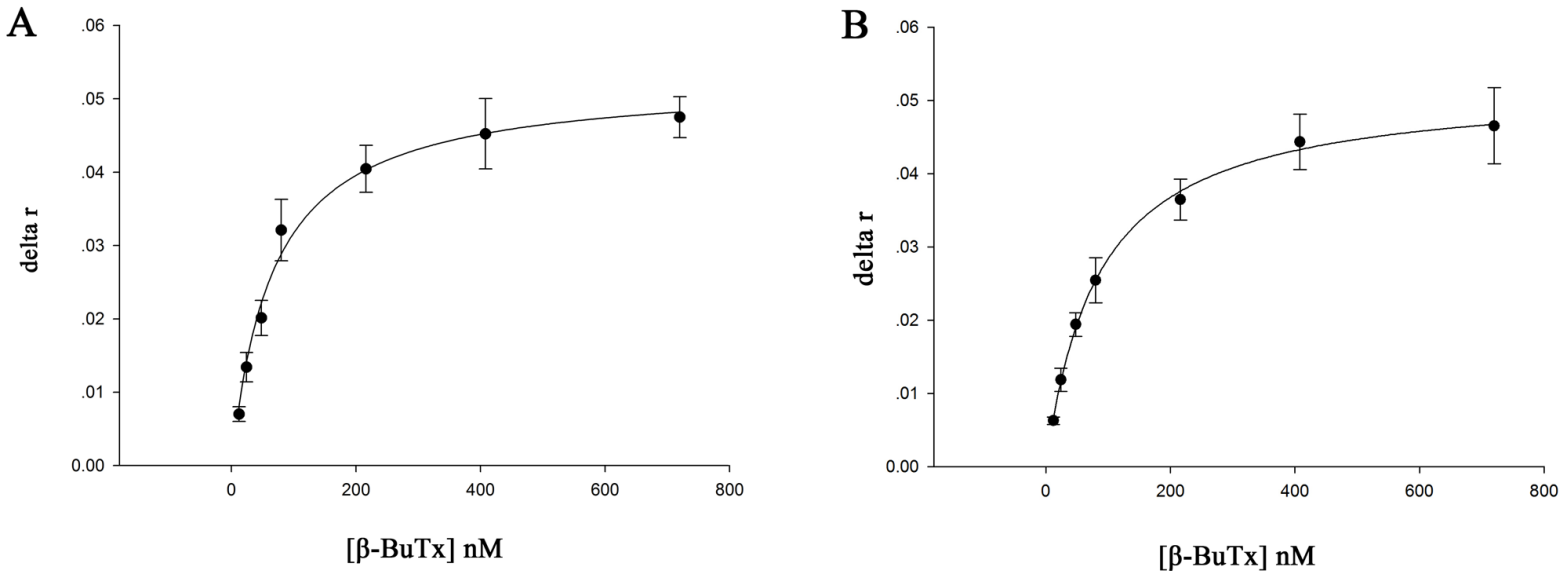
Dissociation constant (K_d_) measurements by fluorescence anisotropy. A) βB-1; B) βB-20. Two nM of fluorescein labeled aptamers were titrated with increasing concentrations of β-BuTx. The anisotropy change (delta r) was the anisotropy of the initial fluorescein labeled aptamer subtracted from the anisotropy value at each β-BuTx concentration. Non-linear regression analysis revealed the K_d_ of βB-1 was 65.9±7.8 nM, and the K_d_ of βB-20 was 83.8±4.3 nM. Error bars represent standard deviations from three repeated experiments.

To evaluate the binding affinities of these sequences, fluorescence anisotropy measurements were conducted for all the selected aptamers. The complete sequences of aptamers were synthesized and labeled with FAM at the 5′-terminal, and then titrated with different concentrations of β-BuTx. When the fluorescently labeled aptamer bound to the target protein, the molecular weight of the complex increased which hindered the rotation diffusion rate of the labeled fluorescein and increased polarization, resulting in a significant anisotropy change of the aptamer. Figure 2 shows the change in anisotropy of aptamers βB-1 and βB-20 versus β-BuTx concentration. The K_d_s were analyzed by using a single-site ligand binding model and those of βB-1 and βB-20 were found to be 65.9±7.8 nM and 83.8±4.3 nM, respectively. The K_d_s of βB-19 and βB-32 were much higher. The K_d_ of βB-19 was 530±58 nM, while that of βB-32 was 995±138 nM (Figure S3). It indicated that the affinity of βB-19 and βB-32 was much lower than that of βB-1 and βB-20.

**Figure 3:**
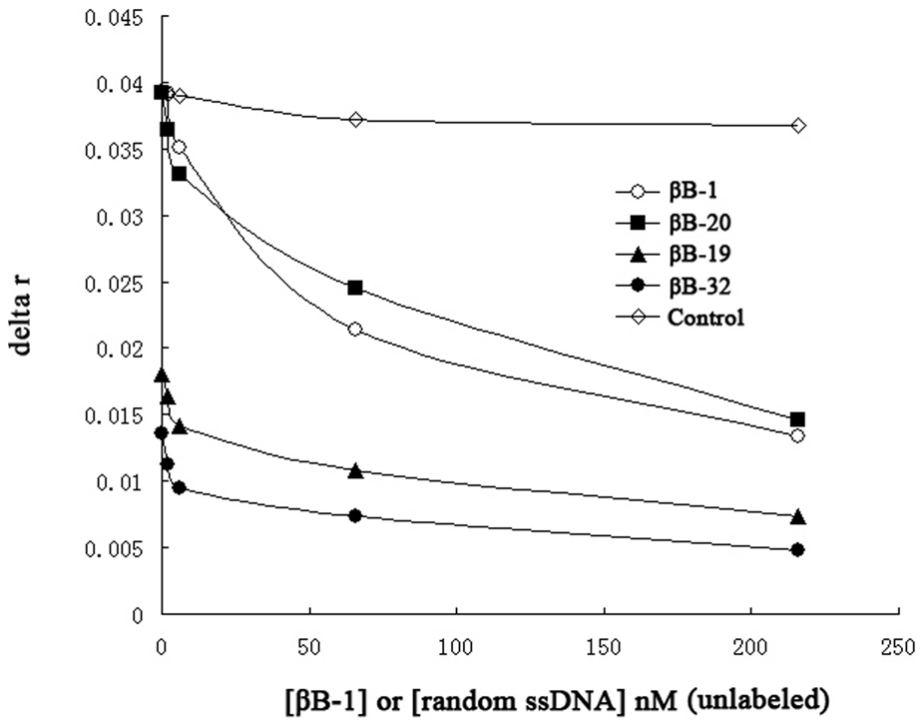
Determination of the binding sites of selected aptamers by competitive anisotropy assay. Two nM of fluorescently labeled aptamer (βB-1, βB-20, βB-19 or βB-32) was firstly incubated with 288 nM of β-BuTx, and then titrated with increasing concentrations of unlabeled aptamer βB-1. Labeled βB-1 was also titrated with unlabeled random ssDNA as control.

In order to compare the binding sites of the selected aptamers, competition assays were performed. Two nM of fluorescently labeled aptamer (βB-1, βB-20, βB-19 or βB-32) was firstly incubated with 288 nM of β-BuTx, and then titrated by unlabeled aptamer βB-1. If the unlabeled βB-1 competes for the same binding site as labeled aptamers, the displacement of the aptamer by βB-1 will result in a decrease in anisotropy [47]. As shown in Figure 3, with increase amount of βB-1, a decrease of fluorescene anisotropy was observed. It indicated that labeled aptamers were displaced by competitor βB-1, suggesting that the binding sites of the four aptamers are the same. Control experiments were done by unlabeled random starting ssDNA library competed with labeled βB-1, and no apparent anisotropy changes were experienced (Figure 3).

### Binding capacity of aptamer βB-1 against related proteins

**Figure 4:**
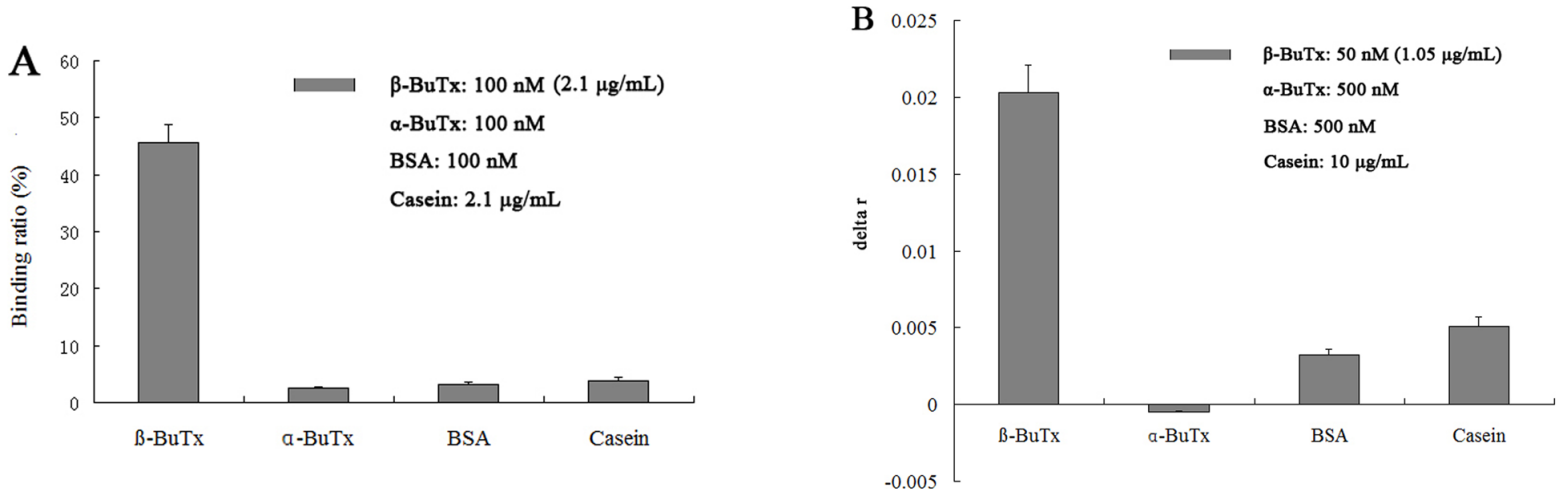
Binding affinity of the aptamer βB-1 to related proteins. A) Ten nM of fluorescein labeled βB-1 was mixed with 100 nM of β-BuTx, BSA, α-BuTx and 2.1 µg/mL of casein, respectively, and then nitrocellulose filter binding assay was performed to calculate the amount of βB-1 bound to tested protein; B) Two nM of fluorescently labeled aptamer βB-1 was mixed with 50 nM of β-BuTx and 500 nM of α-BuTx and BSA, and 10 µg/mL of casein, respectively, the anisotropy changes of βB-1 were determined. Values represent means ± standard errors.

The aptamer βB-1 with highest affinity against β-BuTx was chosen for assessment of its binding capacity against related proteins. We compared the ability of βB-1 to bind β-BuTx, BSA, casein and α-BuTx. BSA and casein were the proteins that used for blocking during selection process, and α-BuTx is a postsynaptic neurotoxin from *B. multicinctus* venom. The results from nitrocellulose filter binding assay (Figure 4A) showed that about 45% of βB-1 bound to β-BuTx, and less than 4% bound to other three proteins when 10 nM of βB-1 incubated with 100 nM of β-BuTx, BSA, α-BuTx and 2.1 µg/mL of casein, respectively. The affinity of βB-1 to the four proteins was also measured via fluorescene anisotropy measurements. As shown in Figure 4B, when 2 nM of βB-1 incubated with 50 nM of β-BuTx and 10 fold excess of other three proteins, the anisotropy change caused by β-BuTx was significantly higher than those by other proteins. The results confirmed that the binding between βB-1 and β-BuTx was not only highly affinitive, but also highly selective.

### Aptamer βB-1 specifically recognize *B. multicinctus* venom

**Figure 5:**
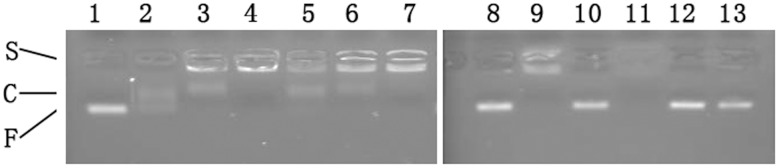
EMSA analysis of the binding of aptamer βB-1 to β-BuTx and five snake venoms. One hundred nM of βB-1 was incubated at RT for 40 min with 240 nM (2), 480 nM (3), 960 nM (4) of β-BuTx, 15 µg/mL (5), 30 µg/mL (6), 50 µg/mL (7 and 9) of *B. multicinctus* venom, and 50 µg/mL of venom of *B. fasciatus* (10), *Naja Naja Atra* (11), *D. acutus* (12), and *G. brevicaudus* (13). Lane 1 and Lane 8: no protein or venom added. F: free DNA; C: DNA/protein complex; S: DNA stuck in well.

Snake venoms contain a highly complex mixture of proteins, enzymes, and various other substances with toxic and lethal properties. Moreover, some snake venoms are reported to contain enzymes that have DNase activity which can hydrolyze DNA [48], [49]. To determine whether the high concentration of other proteins presented in the snake venoms would have an influence on the aptamer binding or hydrolyze aptamer DNA, the reaction between βB-1 and five snake venoms, *B. multicinctus*, *B. fasciatus*, *Deinagkistrodon acutus*, *Gloydius brevicaudus* and *N. Naja Atra*, was investigated by EMSA. *B. multicinctus* and *B. fasciatus* are from the same genus of family Elapidae, *D. acutus* and *G. brevicaudus* are from two different genera of family Viperidae, and *N. Naja Atra* is from genus Naja of family Elapidae. *B. multicinctus* venom contains β-BuTx, while the four other snake venoms do not. As shown in Figure 5, aptamer/protein complex can be seen on the gel when 100 nM of βB-1 mixed with 240 nM and 480 nM of β-BuTx, or with 15 and 30 µg/mL of *B. multicinctus* venom. While the concentration of β-BuTx and *B. multicinctus* venom increased to 960 nM and 50 µg/mL, respectively, the aptamer DNA stuck in the sample well completely. When *N. Naja Atra* venom was tested at the concentration of 50 µg/mL, aptamer DNA was totally hydrolyzed. However, the other three snake venoms did not obviously hydrolyze aptamer βB-1 or bind to it when tested at the same concentration, as there were only free DNA bands on the gel, and the lighteness of the bands were almost the same as the control βB-1 band (Figure 5). The results here showed that the four snake venoms, *B. multicinctus*, *B. fasciatus*, *D. acutus* and *G. brevicaudus* venom, did not apparently hydrolyze aptamer βB-1, and βB-1 only bound to *B. multicinctus* venom but not other structurally or functionally related three snake venoms, even from the same genus.

**Figure 6:**
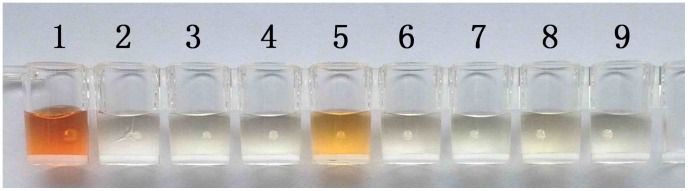
Identification of β-BuTx and *B. multicinctus* venom using aptamer βB-1 via ELAA. Snake venoms and proteins of 10 µg/mL were coated onto ELISA plate wells and incubated with 10 pM biotin-labeled aptamer βB-1, respectively. Then HRP-labeled Streptavidin was added and substrate OPD was used to achieve colorimetric responses. Control well was coated with β-BuTx but no aptamer was added. Well 1: β-BuTx; Well 2: α-BuTx; Well 3: BSA; Well 4: casein; Well 5: *B. multicinctus*; Well 6: *B. fasciatus*; Well 7: *D. acutus*; Well 8: *G. brevicaudus*; Well 9: control.

To further confirm the specificity of βB-1 against β-BuTx and *B. multicinctus* venom, ELAA was performed using the above venoms excluding *N. Naja Atra* venom and proteins β-BuTx, BSA, casein and α-BuTx. Snake venoms and proteins of 10 µg/mL were coated onto ELISA plate wells and incubated with 10 pM biotin-labeled aptamer βB-1, respectively. Then HRP-labeled Streptavidin was added and substrate OPD was used to achieve colorimetric responses. Control well was coated with β-BuTx but no aptamer was added. As shown in Figure 6, the color of wells coated with β-BuTx and *B. multicinctus* venom turned to be brown, while the wells of other proteins and snake venoms, as well as the control well, underwent no obvious optical change. Without the aid of a detection device, one can apparently distinguish β-BuTx from BSA, casein and α-BuTx, and *B. multicinctus* venom from three tested snake venoms. Furthermore, the color of β-BuTx well was deeper than that of *B. multicinctus* venom well. This was because that only part of the *B. multicinctus* venom components was β-BuTx. The results proved that βB-1 bound only to β-BuTx and β-BuTx-containing *B. multicinctus* venom, but not to other proteins or snake venoms. It also confirmed that the binding between βB-1 and β-BuTx was not interfered with by other ingredients presented in the four species of snake venoms.

In conclusion, we have used plate SELEX to successfully isolate DNA aptamers for β-BuTx with nanomolar affinity in 10 rounds of selection. Based on the results of the present study, it demonstrates that the aptamer βB-1 specifically binds to β-BuTx, and can discriminate *B. multicinctus* venom from different species of snake venoms. These results suggest that aptamer βB-1 could be useful in the development of diagnostic assays for snakebites. As aptamers are susceptible to nuclease-mediated cleavage, they are not stable in biofluid samples. Further chemical modifications of the selected aptamer should be made to improve its biostability.

## Supporting Information

Figure S1 **Representative pilot PCR of the 10^th^ round of selection.** Electrophoresis on 3% agarose after 18–26 cycles of PCR amplification. Lane 1: 20 bp DNA Ladder (TaKaRa); Lanes 2–5: 18, 20, 22, 24 and 26 cycles of PCR. The products of 20 cycles of PCR amplification were relatively specific fragments.
(TIF)

Figure S2 **The secondary structures of aptamers βB-1, βB-19, βB-20 and βB-32.**
(TIF)

Figure S3 **K_d_ measurements of βB-19 and βB-32 by fluorescence anisotropy.** A) βB-19, K_d_ = 530±58 nM; B) βB-32, K_d_ = 995±138 nM.
(TIF)

Table S1 **Selection parameters of each SELEX round.**
(DOC)
