# Supplementary figures and images for: Recognition of Bungarus multicinctus Venom by a DNA Aptamer against β-Bungarotoxin
Source: PLoS One. 2014 Aug 21;9(8):e105404. doi: 10.1371/journal.pone.0105404 (PMC4140777; doi:10.1371/journal.pone.0105404)

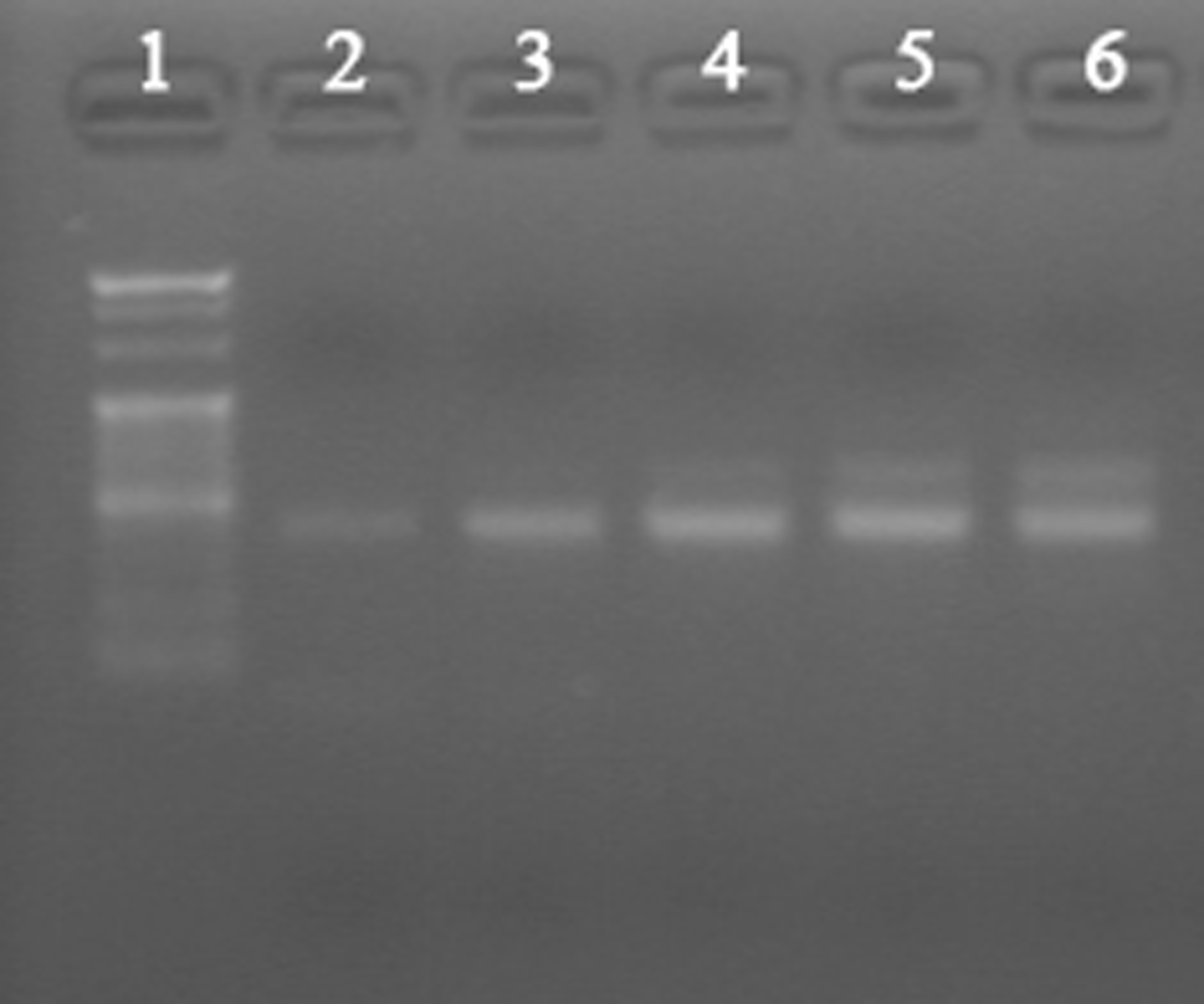

Supplement: Figure S1 — Representative pilot PCR of the 10th round of selection. Electrophoresis on 3% agarose after 18–26 cycles of PCR amplification. Lane 1: 20 bp DNA Ladder (TaKaRa); Lanes 2–5: 18, 20, 22, 24 and 26 cycles of PCR. The products of 20 cycles of PCR amplification were relatively specific fragments. (TIF) [file pone.0105404.s001.tif]

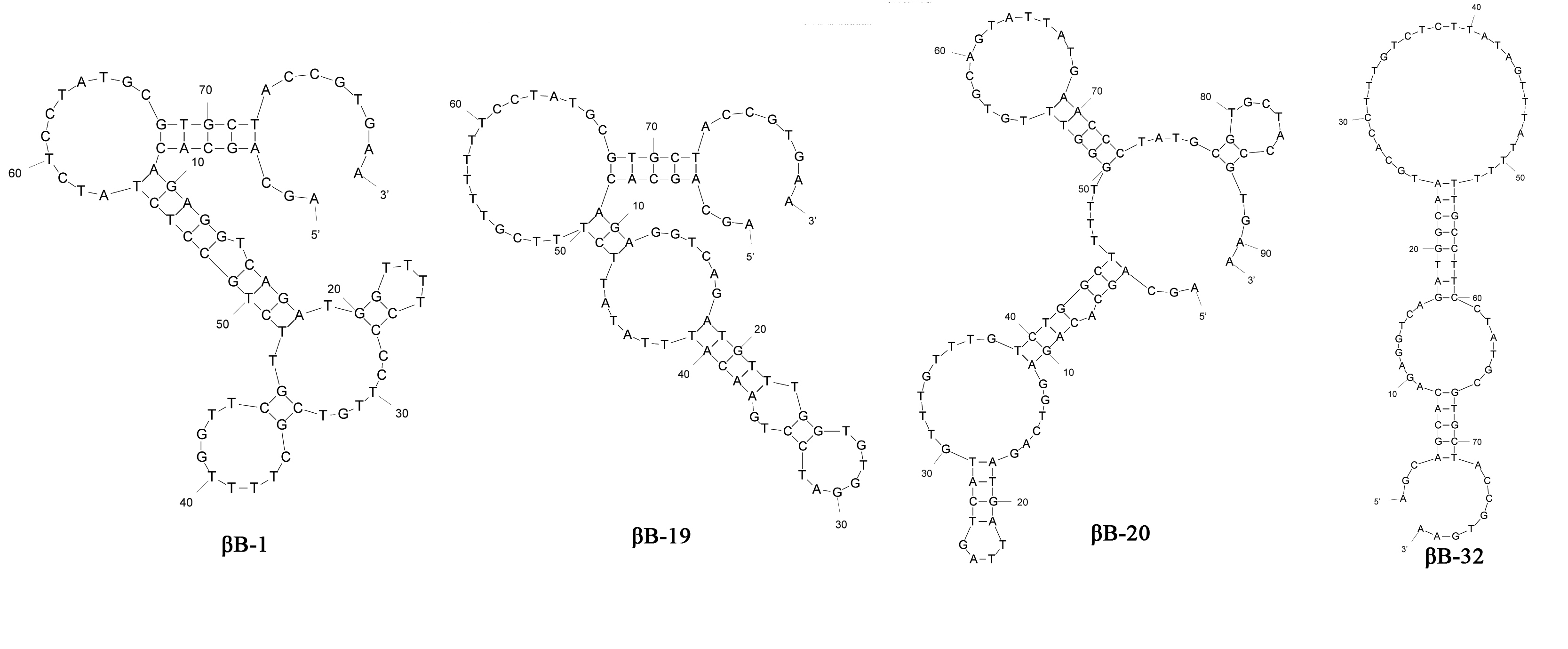

Supplement: Figure S2 — The secondary structures of aptamers βB-1, βB-19, βB-20 and βB-32. (TIF) [file pone.0105404.s002.tif]

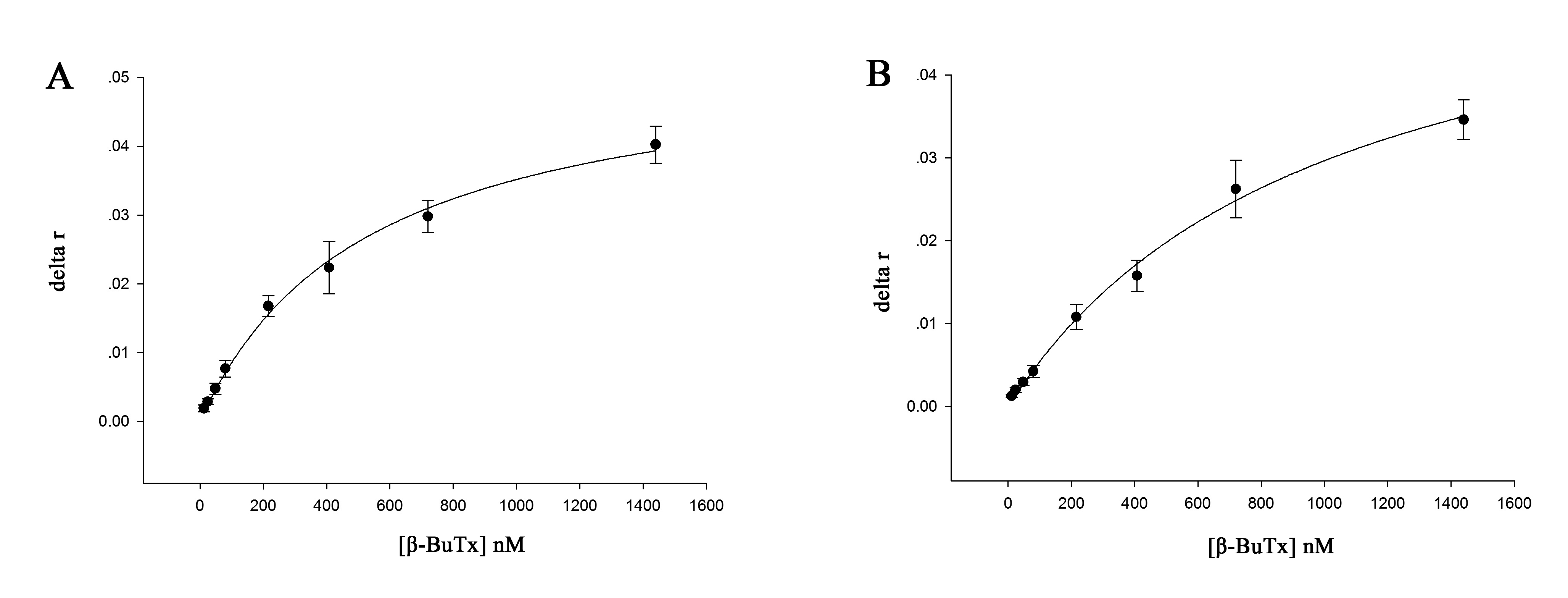

Supplement: Figure S3 — Kd measurements of βB-19 and βB-32 by fluorescence anisotropy. A) βB-19, Kd = 530±58 nM; B) βB-32, Kd = 995±138 nM. (TIF) [file pone.0105404.s003.tif]
