# Supplementary material for: Recognition of Bungarus multicinctus Venom by a DNA Aptamer against β-Bungarotoxin
Source: PLoS One. 2014 Aug 21;9(8):e105404. doi: 10.1371/journal.pone.0105404 (PMC4140777; doi:10.1371/journal.pone.0105404)
Supplement: Table S1 — Selection parameters of each SELEX round. (DOC) [file pone.0105404.s004.doc]

**Table S1.** Selection parameters of each SELEX round.

| SELEX round | β-BuTx /well(μg/mL) | ssDNA/well(pmol) | PCR cycle |
| --- | --- | --- | --- |
| 1 | 10 | 1000 | 19 |
| 2 | 10 | 100 | 20 |
| 3 | 10 | 100 | 19 |
| 4 | 10 | 100 | 17 |
| 5 | 10 | 80 | 18 |
| 6 | 10 | 80 | 17 |
| 7 | 10 | 80 | 21 |
| 8 | 10 | 50 | 20 |
| 9 | 10 | 50 | 23 |
| 10 | 10 | 50 | 20 |
